# Heterologous Expression of a Thermostable Chitinase from *Myxococcus xanthus* and Its Application for High Yield Production of Glucosamine from Shrimp Shell

**DOI:** 10.3390/foods10112808

**Published:** 2021-11-15

**Authors:** Yongmei Lyu, Feng Zheng, Chuanxing Qiu, Meng Wang, Dujun Wang, Xiaoyang Zhang, Josef Voglmeir, Li Liu, Xiaohong Yu

**Affiliations:** 1 School of Marine and Bioengineering, Yancheng Institute of Technology, Yancheng 224051, China; lyu.yongmei@ycit.edu.cn (Y.L.); xq19516560569@163.com (C.Q.); wangdj@ycit.edu.cn (D.W.); zhang.xiaoyang@ycit.edu.cn (X.Z.); 2 Glycomics and Glycan Bioengineering Research Center (GGBRC), College of Food Science and Technology, Nan-jing Agricultural University, Nanjing 210095, China; 2016108004@njau.edu.cn (F.Z.); 2016208028@njau.edu.cn (M.W.); josef.voglmeir@njau.edu.cn (J.V.)

**Keywords:** glucosamine, chitinase, *M. xanthus*, chitin, enzymatic cascades

## Abstract

Glucosamine (GlcN) is a widely used food supplement. Hence, enormous attention has been concerned with enzymatic production of GlcN owing to its advantage over a chemical approach. In this study, a previously unstudied chitinase gene (MxChi) in the genome of *Myxococcus xanthus* was cloned, expressed in recombinant soluble form and purified to homogeneity. TLC-, UPLC-, and microplate-reader- based activity tests confirmed MxChi hydrolyzes colloidal chitin to chitobiose as sole product. The optimal catalytic pH and temperature of MxChi was identified as 7.0 and 55 °C, respectively. MxChi exhibited 80% activity after 72 h incubation at 37 °C. The site-directed mutagenesis revealed that the amino acids D323A, D325A, and E327A of MxChi were in the DXDXE catalytic motif of GH18. When coupled with β-N-acetylhexosaminidase (SnHex) and deacetylase (CmCBDA), the enzyme allowed one-pot extraction of GlcN from colloidal chitin and shrimp shell. The optimal condition was 37 °C, pH 8.0, and 1/3/16.5 (MxChi/SnHex/CmCBDA), conducted by orthogonal design for the enzymatic cascades. Under this condition, the yield of GlcN was 26.33 mg from 400 mg shrimp shell. Facile recombinant in *E. coli*, robust thermostability and pure product herein makes newly discovered chitinase a valuable candidate for the green recycling of chitin rich waste.

## 1. Introduction

According to an estimate, approximately 6–8 million tons of crab, shrimp, and lobster shell waste is produced annually around the world. Most of it is directly dumped into the sea or landfill [1], used for composting, and to produce fertilizers and animal feed [2]. However, in recent years this type of waste has begun to attract more attention as a source of raw material with remarkable benefits [3]. It is well known that in addition to protein and calcium carbonate, shells contain chitin, the second most easily available polysaccharide after cellulose. Chitin, an insoluble linear biopolymer of N-acetyl D-glucosamine (GlcNAc), is also found in yeasts, fungi and arthropod exoskeleton [4]. The content of chitin reaches 15–40% dry weight of the shells of crustaceans [5]. The potential value of such shells in the chemical or biotechnology industry field is normally neglected. 

Chitin has several applications when converted into its deacetylated derivative chitosan, chitooligomers, and monomers, such as glucosamine (2-amino-2-deoxy-D-glucose, GlcN) [6], which are high-added-value compounds in food, pharmaceutical industries, textile medical industries, and agriculture [7,8,9]. For example, GlcN has been widely used as a dietary supplement for osteoarthritis treatment [10,11]. Chitin can be transformed to chitosan chemically or enzymatically [12]. Conventional chemical processes involve harsh acidic hydrolysis or use of alkali and are commonly used to produce chitosan at commercial scale [13]. However, considering the environment pollution, an environmentally friendly process of chitin processing that results in a high purity and quality product is highly desirable. Thus, enzymatic degradation of chitin has gained a great deal of attention in recent years because of its green characteristics [14,15]. 

Chitinase (EC 3.2.1.14), a glycoside hydrolase (GH), hydrolyzes the glycosidic bond of chitin generating mono-or oligomers [16] and is one of the enzymes utilized for biotransformation of chitin to chitosan. Since their discovery, chitinases, including glycosyl hydrolases 18 and 19 families based on their amino-acid sequences and catalytic mechanisms in CAZy database [17], have been found in different organisms in all kingdoms [18,19,20]. As an enzyme tool, chitinase plays an important role in the degradation of chitinous waste [21], and is used in the food, medicine, and agriculture industry for the biocontrol of pathogenic fungi [22]. Notably, chitinases derived from bacteria have the potential for industrial application due to their excellent properties and simple genetic information [18], and thus, the quest for bacterial chitinases is of high importance. 

*Myxococcus xanthus* is a Gram-negative ubiquitous soil bacterium that can be classified as proteolytic cellulolytic and chitinolytic depending on what substrate it grows in [23]. Chitinases and *β*-1,6-glucanases from *M. xanthus* were shown to be essential weapons for the efficient predation of fungi [24]. Although these enzymes were studied as attractive candidates for the control of pathogenic fungi and harmful insects [25], no chitinase from *M. xanthus* has been heterogeneously expressed and characterized. In this report, we successfully cloned, expressed, biochemically characterized, performed enzymatic cascades optimization, and tested a potential application of a GH18 chitinase from *M. xanthus*. Our results show that a newly discovered chitinase from *M. xanthus* could be a promising candidate for the eco-friendly bioconversion of chitin waste to its highly valued products.

## 2. Materials and Methods

### 2.1. Materials

*M. xanthus* DK1622 (DSM 435) was obtained from the German Collection of Microorganisms and Cell Culture (DSMZ). *Escherichia coli* Mach1 cells (Life Technologies, Shanghai, China) were used for plasmid amplification and manipulation. *E. coli* strain BL21 (Invitrogen, Shanghai, China) was used as the expression enzyme. The pET30a (+) plasmid was used as vector for the heterologous overexpression. Primestar DNA polymerases were purchased from Takara (Dalian, China). Restriction endonucleases (NdeI, XhoI, and DpnI) and T4 ligases were purchased from Thermo Fisher Scientific (Shanghai, China). The Plasmid Extraction Kit and the DNA Gel Purification Kit were from Axygen (Beijing, China). DNA primers were synthesized by GenScript (Nanjing, China). Thin-layer chromatography (TLC) was performed using Merch 60 F254 precoated silica gel aluminium plates. Substrates and products were visualized by exposure to a solution of diphenylamine (0.1 M), phosphoric acid (10% *v*/*v*) and aniline (2% *v*/*v*), in acetone (DPA). Kanamycin and isopropyl-*β*-D-thiogalacto-pyranoside (IPTG) were purchased from Sigma-Aldrich Chemical Co. (St. Louis, MO, USA). All other reagents used in this study were sourced from local chemical suppliers.

### 2.2. Colloidal Chitin Preparation, Gene Amplification and Plasmid Construction

In order to make the chitin more soluble, 5 g of chitin was mixed with 150 mL of 85% phosphoric acid and stirred at 4 °C for 5 h. 50 mL 50% cold ethanol was added and stirred to precipitate the chitin, followed by centrifugation at 12,000× *g*, 4 °C for 10 min. The precipitate was washed with distilled water until the pH reached neutral and re-suspended in distilled water to a final concentration of 20 mg/mL.

Genomic DNA was extracted by incubating bacterial cells in lysis buffer (50 mM Tris-HCl, 500 mM EDTA, 10% SDS, 1% Triton X-100, 50 mM NaCl, and 0.2 µg/µL proteinase K, pH 8.0) at 55 °C for 3 h according to the method reported by Mahuku [26]. The primers of DNA encoding chitinase from *M. xanthus* (UniProt ID: Q1D885) were designed using the genomic data provided by Pathosystems Resource Integration Center (PATRIC) [27] and consisted of the following base pairs: 5′-CATATGGTGCCGTCGTCTCACGTTCG-3′ (F1, containing an underlined NdeI restriction site) and 5′-CTCGAGGCGCAGCTTCCGTGCCAG-3′ (F2, containing an underlined XhoI restriction site). The DNA was amplified by polymerase chain reaction (PCR) with Primestar DNA polymerase. PCR reaction product was separated by an agarose gel and purified using the DNA gel purification kit, and was ligated after restriction digest into pET30a, which had been predigested with the same restriction endonucleases. By using these restriction sites, the open reading frame of construct vector carried an N-terminal Histag/thrombin/S-tag configuration followed by an enterokinase cleavage site. The ligation mixture was transformed into *E. coli* Mach1 T1 cells and plated on Luria–Bertani (LB) agar containing 50 mg/L kanamycin for selection. Colonies that were confirmed to contain the expected plasmid construct by Sanger DNA sequencing (Sangon Biotech, Shanghai, China), were selected for further experiments. Active site mutants were carried out using the standard protocol for QuikChange Site-Directed Mutagenesis (Stratagene) with the primer pairs listed in Table S1. Plasmid extraction, digestion with restriction enzymes, DNA ligation, purification, and transformation procedures were performed using molecular biological standard protocols.

### 2.3. Expression and Purification of Heterologous MxChi

The recombinant plasmids pET30a/MxChi were transferred into *E. coli* BL21(DE3) competent cells and plated on LB agar supplemented with 50 mg/L kanamycin. A single colony was picked and inoculated in a 5 mL culture at 37 °C overnight. 1 mL of the cell suspension was then transferred into 400 mL LB medium (37 °C, 200 rpm). MxChi overexpression was induced at the early exponential phase (OD_600_ reached 0.6–0.8), with 1 mM IPTG, and the cultivation was continued for 20 h at 18 °C, 200 rpm. The cells were harvested by centrifugation (4000× *g*, 15 min), and the cell pellets were suspended in 10 mL lysis buffer (50 mM Tris, 100 mM NaCl, 1% Triton X-100 and 1 mM PMSF, pH 8.0) and disrupted by sonification for 20 min. The cell debris was then separated by centrifugation at 12,000× *g*, 4 °C for 20 min. The resulting supernatant containing MxChi was loaded onto a Ni^2+^-nitrilotriacetate (Ni^2+^NTA) agarose affinity chromatography column (2 mL bed volume, Qiagen, Hilden, Germany). The column was washed with washing buffer (100 mM NaCl, 50 mM Tris, 10 mM imidazole, pH 8.0) to remove unbound proteins. Recombinant MxChi were eluted using an elution buffer (100 mM NaCl, 50 mM Tris, 500 mM imidazole, pH 8.0). Fractions showing the high UV absorbance at 280 nm were pooled, desalted by gel-filtration chromatography using prepacked PD-10 cartridges (GE Healthcare, Chicago, IL, USA) with 50 mM Sodium phosphate buffer (pH 8.0), frozen in liquid nitrogen, and preserved at −80 °C until use. The protein expression, cell lysis and purified proteins were subjected to SDS-PAGE after Coomassie Brilliant G-250 staining [28]. The protein concentration was determined using the quantification method of a Bradford protein assay (Sangon Biotech) and bovine serum albumin (BSA) as a standard. 

### 2.4. Enzyme Activity Assay and Analytical Methods

Activity screens of recombinant MxChi were performed using a laser particle size analyzer, TLC method and an ultra high-performance liquid chromatography (UPLC) method described previously [29,30], with some changes. Unless otherwise indicated, the enzyme reaction mixture (100 µL) contained 5 mg/mL of colloidal chitin, 50 mM of Na_2_PO_4_ buffer (pH 8.0) and recombinant enzyme (100 µg/mL). Enzyme-free or substrate-free mixtures were used as negative controls. The assays were carried out at 37 °C for 30 min and then quenched by adding 100 µL of chloroform. After centrifugation, an aliquot of the upper-phase was used for the production determination. For TLC analysis, samples (2 µL) were spotted on the activated silica gel plate and allowed to develop in a solvent containing n-butanol/ethanol/water mixture (5/3/2, *v*/*v*/*v*) as the eluent to monitor the reaction product. Compounds were revealed using an aniline-based stain. The staining solution consisted of diphenylamine (5.91 mmol), phosphoric acid (5 mL), aniline (10.75 mmol), and acetone (50 mL), and was followed by heating at 95 °C for 10 min prior to analysis.

UPLC was performed on a Shimadzu Nexera HPLC system (Shimadzu Corporation, Tokyo, Japan) consisting of an LC-30 AD pump equipped with a low-pressure gradient mixing unit, an SIL-30 AC autosampler detector, and a fluorescence detector (RF-20Axs). The analytes were dried before labelling by fluorescent reagent 2-aminobenzamide (2-AB) using the SIGNAL^TM^ 2-AB labelling kit (Prozyme, Pensacola, FL, USA) according to the manufacturer’s instructions and separated on an Acquity BEH Glycan column (Waters, 1.7 μm, 2.1 × 150 mm) with a 0.5 mL/min flow rate at 60 °C. The effluents were monitored by the fluorescence detector (excitation: 330 nm, emission: 420 nm). Ammonium formate in water (50 mM, pH 4.5) and 100% acetonitrile were used as mobile phase A and mobile phase B, respectively. A linear gradient of 95–78% of B was applied from 0 to 6 min. B was then decreased to 56% at 44.5 min, then further decrease to 0% over 3 min and held at 0% for another 2 min. B was then increased to 95% in 2 min and the column was equilibrated with the initial conditions for 7 min. One unit of chitinase activity was defined as the amount of enzyme required to produce 1 μmol of chitobiose per min. 

The microscopic changes of MxChi treated colloidal chitin were monitored using a laser particle size analyzer (Malvern Master Sizer, Malvern Instruments, Ltd., Worcestershire, UK). The time-interval of MxChi reaction was 1 h, 2 h, 6 h, and 12 h. inactive mutant MxChi was a negative control.

In addition, an alternative approach of detecting the activity of mutants was Gongo Red dye, a modified method from Raval et al. [31]. Briefly, 50 mM Na_2_HPO_4_/NaH_2_PO_4_, 5 mg/mL colloidal chitin, and 15 g/L agarose in pH 7.0 was autoclaved and poured in the petri-dish. The holes to add the purified wild-type and mutant enzyme solutions were punched. The petri-dish containing enzymes was incubated at 37 for overnight. It was subsequently stained with 0.1% of Congo red dying solution followed by washing with 1 M NaCl. The lytic zone was then visualized.

### 2.5. Chitinase Biochemical Characterization

To determine the biochemical parameters of MxChi, a colorimetric assay based on the derivatization of free amine with o-phthalaldehyde (OPA) was used, a method reported previously by Lv et al. [29,32]. The assay design is based on the coupling of the chitinase, activity beta-hexosaminidase, and GlcNAc deacetylase [32], which catalyzes the formation of chitobiose from chitin, the formation of GlcNAc from chitobiose, and the formation of GlcN from GlcNAc, respectively (Figure S3). Briefly, typical chitinase activity reaction mixture was quenched by heating to remove the chitinase, then *β*-N-acetylhexosaminidase (SnHex) was added for overnight to fully convert the product of chitinase to GlcNAc, GlcNAc deacetylase CmCBDA was used at last. The final product GlcN quantity, which is positive correlation with MxChi activity, was monitored continuously with OPA derivatization at 340 nm on a microplate-reader (Thermo Scientific Multiskan, Waltham, MA, USA) with a temperature-controlled cuvette holder. Pure GlcN was used as the reference material for quantification. 

The pH activity profile of recombinant MxChi was determined by incubating reaction mixtures in different buffers covering various pH ranges (Citric acid/Na_2_HPO_4_: 3.0, 4.0, 5.0, 6.0, and Na_2_HPO_4_/NaH_2_PO_4_: 7.0 and 8.0, and sodium carbonate: pH 9.0 and 10.0) at 37 °C for 30 min. The pH was charged back to 8.0 for subsequent SnHex and CmCBDA hydrolyzing. The temperature-dependent activity profile of recombinant MxChi was determined by incubating the reaction mixtures at various temperatures between 4 °C to 70 °C for 30 min. The thermal stability was determined by the pre-incubation of recombinant MxChi at 30 °C, 37 °C, 45 °C, 50 °C, 55 °C, and 60 °C for various time intervals (0 h, 12 h, 24 h, 36 h, 48 h, 60 h, and 72 h. The effect of metal ions on recombinant MxChi was determined by adding 2 mM of different metal ions (CuCl_2_, CaCl_2_, MgCl_2_, MnCl_2_ and NiCl_2_) or EDTA to the reaction mixtures. The reaction mixture without enzyme or substrate was anegative control. Colloidal chitin, with a final concentration ranging from 0.05 to 10 mg/mL, was used to investigate the kinetic constants *K_m_* and *V_max_*.

### 2.6. Enzymatic Synthesis of GlcNAc from Colloidal Chitin 

Reaction mixtures (1 mL) containing 5 mg/mL of colloidal chitin, 50 mM of Na_2_HPO_4_ buffer (pH 8.0) and recombinant MxChi (100 µg/mL), and SnHex (100 µg/mL) were carried out at 37 °C. The time-course kinetic studies were performed and represented in the form of activity vs time graph showing production levels of GlcNAc at different time intervals (1 h, 2 h, 6 h, and 12 h). The samples were quenched by heating at 95 °C for 5 min. Inactive mutant MxChi or SnHex mixtures were used as negative controls with the incubation time of 12 h. TLC and OPA based colorimetric assay were used to monitor the products. Pure GlcNAc and chitobiose were used as the standards.

### 2.7. Enzymatic Synthesis of GlcN from Colloidal Chitin and Shrimp Shell

Fresh shrimp (*Macrobrachium nipponense*) meat was removed. Shrimp shell, which was frozen with liquid nitrogen, was pulverized, dialyzed, and freeze dried. A typical reaction was performed on a 1 mL-scale and consisted of 40 mg shrimp shell or 5 mg colloidal chitin, 100 µg of MxChi, 100 µg of SnHex, and 500 µg of CmCBDA in Na_2_HPO_4_ buffer (50 mM, pH 8.0) at 37 °C for different time intervals. The samples containing inactive mutants of three enzymes were used as negative controls with the incubation time of 12 h. TLC and OPA based colorimetric assay were performed as described above. Pure GlcNAc and GlcN were used as the standards.

### 2.8. Orthogonal Design for GlcN Production from Shrimp Shell

Based on the typical reaction condition, the single-factor experiments were carried out. Reaction pH, reaction temperature, and enzyme amount ratio were chosen as the three important factors. pH 5.0, 6.0, 7.0, 8.0 and 9.0 were chosen to test the optimum pH of enzymatic synthesis of GlcN from shrimp shell. The optimum temperature of enzyme cascades was determined by incubating the reaction mixtures at different temperature from 32 °C to 62 °C. The ratio of MxChi to SnHex (1:1, 1:2, 1:3, 1:4, or 1:5, *w*/*w*) was tested. Based on the optimal ratio of MxChi to SnHex, the optimum amount ratio of MxChi/SnHex/CmCBDA was further conducted. Then, an orthogonal design L9 (3^3^) was used in this experiment. The evaluation criterion of enzymatic cascades effect is based on the maximum GlcN yield from per 40 mg shrimp shell. (Table 1). OPA based colorimetric assay was performed as described above. An enzyme cocktail in optimized condition was used for converting 400 mg shrimp shell to GlcN. The reaction was carried out at optimized condition with the scale of 10 mL. The concentration of GlcN was determined as stated above.

### 2.9. Phylogenetic Analysis Homology Modeling 

Putative functions were inferred using the basic local alignment search tool (BLAST) (http://www.ncbi.nlm.nih.gov/BLAST (accessed on 20 May 2020)). The neighbor-joining method in the molecular evolutionary genetic analysis (MEGA) software package, version 6.0 (http://www.megasoftware.net/ (accessed on 16 June 2021)), was used to construct a phylogenetic tree. The theoretical molecular mass of the deduced MxChi protein sequence was calculated using the Compute pI/Mw tool on the ExPASy proteomics server (available at http://expasy.org/tools/pi_tool.html (accessed on 16 June 2021)). The three-dimensional structure of chitinase from *B. thuringiensis* (PDB, ID: 6BT9) with 41.9% sequence identity to MxChi was selected as the template for homology modeling by utilizing the MODELLER homology software (Version 9.17) [33]. The image of MxChi was created using PyMOL software (Version 2.3.0). The sequence alignments and phylogenetic relationship between chitinases were performed using the MUSCLE and PhyML, respectively, the online tools were provided by Dereeper et al. [34].

### 2.10. Statistical Analysis 

The data represent the means ± standard deviations (SD) from independent triplicates. Statistical analysis was performed using EXCEL software. The graphs were drawn using GraphPad Prism7 software. 

## 3. Results and Discussion

### 3.1. Cloning and Homology Analysis of the MxChi Gene

**Figure 1:**
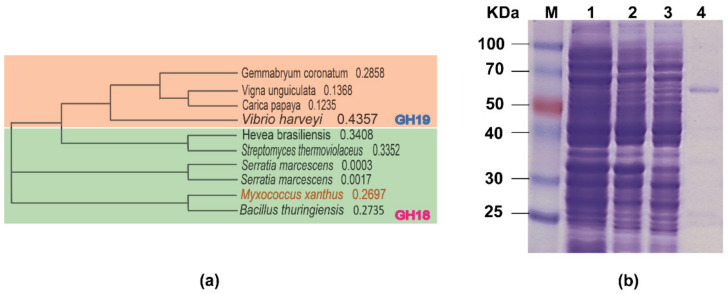
(**a**) Phylogenetic relationship of functionally characterized chitinases. (**b**) SDS-PAGE analyses of recombinant MxChi at various stages of expression and purification. M-protein marker; 1-cell pellet before induction; 2-cell pellet after induction with IPTG; 3-supernatant after cell lysis; and 4-purified protein.

The full-length open reading frame (ORF) encoding a putative chitinase candidate in *M. xanthus* consisting of 1731 bp base pair was successfully amplified and ligated into the expression vector pET30a (Figure S1). No signal peptide was predicted in the chitinase sequence. As the recombinant MxChi contained a fused hexa-histidine tag at the C-terminus, nickel chelation affinity purification was applied to isolate the target protein. The homology search revealed that MxChi closely showed the highest similarity with characterized GH18 enzyme family members, and it has low to moderate sequence similarity to previously known chitinase, reaching 41.9% identity with chitinase from *Bacillus thuringiensis* (UniProt ID: Q81IF9), 30.8% identity with *Serratia marcescens* (UniProt ID: P11797) [35], 30.5% identity with *Streptomyces thermoviolaceus* (UniProt ID: D6K143) [36], and 28.1% identity with *Hevea brasiliensis* (UniProt ID: P23472). Domain structure analysis revealed that MxChi contained a GH18 chitinase domain. A phylogenetic comparison of previously characterized chitinases, shown in Figure 1a, belonging to the known chitinase CAZy families and analysis by Conserved Domain Search Service clearly confirmed that MxChi belongs to the GH18 family.

### 3.2. Recombinant Protein Expression and Purification

**Figure 2:**
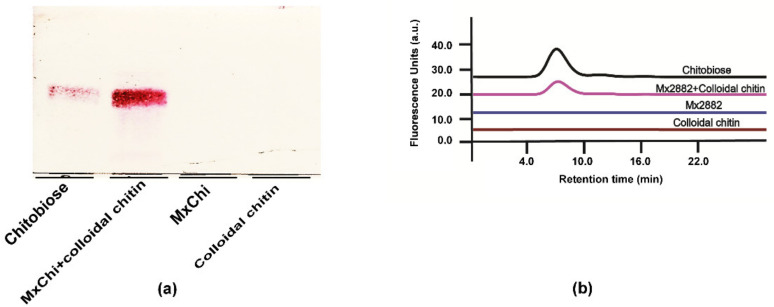
Chitinase activity of recombinant MxChi. (**a**) TLC chromatogram of enzyme reaction product using colloidal chitin as the substrate. The sample without substrate or MxChi was a negative control. TLC was developed with a mobile phase containing n-butanol:ethonal:water at a ratio of 5:3:2 (*v*/*v*/*v*) and were stained with DPA. (**b**) UPLC- based assay for chitinase activity screening with colloidal chitin as substrate. The samples were dried under vacuum and fluorescence labelled with 2-AB derivatization.

The expression and purification of MxChi was evaluated by SDS-PAGE under denaturing conditions (Figure 1b). The recombinant form of MxChi gene was successfully expressed in soluble status. The observed predominant single band of the purified protein showed an apparent molecular weight of approximately 60 kDa (Figure 1, lane 4), which is in good agreement with the expected molecular weight (61 kDa). Combined with UPLC, TLC and particle size analyzer method, the hydrolysis activity of chitinase MxChi were confirmed, and that chitobiose was the only product hydrolyzed from colloidal chitin, indicating that MxChi was a product specific chitinase (Figure 2 and Figure S2). It has similar catalytic mode with the chitinase from *Chitiniphilus shinanonensis* [37], while enzymatic deconstruction of chitin to different chitooligosaccharides by chitinases were well reported [16,38]. Chitobiose has been found to be helpful in agricultural industries and pharmaceutical industries, such as to improve T2D-related metabolic disorders [39] and to act as a bio-stimulator for plant growth [40].

The concentration of the purified MxChi was 2.60 ± 0.37 mg/mL with a specific activity of 320.55 ± 9.73 U/mL. The production of MxChi was 32.22 ± 3.82 mg per liter culture. Previous studies showed that the chitinase production of ChiEn1 from *Coprinopsis cinerea* was 0.15 U/mL, 0.05 g/L [16], PbChi70 from *Paenibacillus barengoltzii* was 2.14 U/mL, 0.17 g/L [41], and Cmchi1 from *Chitinolyticbacter meiyuanensis* SYBC-H1 was 1.98 U/mL, 0.52 g/L [42]. These data demonstrated that MxChi production was higher than many chitinases from other species.

### 3.3. Enzymatic Properties of MxChi

**Figure 3:**
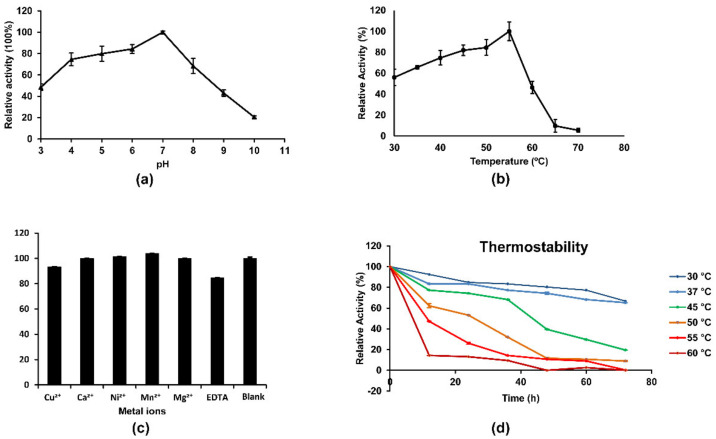
Biochemical characterization of MxChi. (**a**) Effect of pH on activity of purified recombinant MxChi. (**b**) MxChi activity at various incubation temperatures. (**c**) Impact of metal ions and EDTA on the enzymatic activity of MxChi. (**d**) Thermal stability of MxChi using multiple pre-incubation times and temperatures. The error bars represent the standard deviations calculated from three independent experiments.

Chitinases have found diverse industrial, pharmaceutical, and agricultural applications, including chitin rich wastes degradation [43], diseases [20], biocontrol against phytopathogenic fungi and insects [4], and so on, which made scientists particularly interested in discovering new chitinases, while a satisfactory recombinant form with the robust chitinase properties is difficult to obtain. In this study, high-throughput manner by microplate-reader in a coupled enzymatic assay was used to explore the properties of MxChi, which was based on the reported assay by Wang et al. and Lv et al. (Figure S3) [29,32]. The purified MxChi was most active at pH 7.0 in phosphate buffer (Figure 3a), and it was stable within pH 4.0–8.0 as it retained more than 70% of its maximal activity, indicating that the enzyme is a neutral chitinase. Most other bacterial chitinases are also optimally active at neutral pH 6.0–7.0 [37,44,45], while some chitinases were more active at acidic condition, such as the chitinases from *Paenibacillus xylanexedens* Z2-4 (pH 4.5) [25], *Penicillium oxalicum* k10 (pH 5.0) [38], and *Paenibacillus pasadenensis* CS0611 (pH 5.0) [46]. 

The optimal temperature of MxChi was found to be 55 °C (Figure 3b), which is comparable to those of some chitinases from bacteria *P. barengoltzii* [41], *C. shinanonensis* [37], and higher than those of many chitinases such as those from *Sanguibacter antarcticus* (37 °C) [47], *Trichoderma asperellum* PQ34 (45 °C) [22], and *S. maltophilia* (40 °C) [45]. MxChi was especially stable below 37 °C (Figure 3d), and 80% of its maximum activity was left after incubation at 37 °C for 72 h, indicating that MxChi was a robust-stable enzyme. The addition of metal ions (Ca^2+^, Cu^2+^, Mg^2+^, Mn^2+^, and Ni^2+^ in their chloride forms) or EDTA didn’t significantly reduce the activity of the enzyme (Figure 3c). The fact that EDTA in the reaction mixture did not inhibit the activity of MxChi confirms that metal ions are not required in the catalytic mechanism of the enzyme. Even though some other characterized chitinases were reported to be sensitive to various metal ions, no obvious effect was detected on the activity of MxChi within the experimental range. For example, chitinases from *Stenotrophomonas rhizophila* G22 were inhibited by Cu^2+^ [48]. The activity of SaChiA4 was inhibited by Cu^2+^ and EDTA [49]. The chitinase from *Serratia plymuthica* was stimulated by 120%, and 240% in the presence of Ca^2+^ or Mn^2+^ and inhibited by 80% in the presence of Cu^2+^ [5].

The kinetic constants *K_m_* and *V_max_* of MxChi were determined to be 0.98 mg/mL and 12.23 μg/(min·mL), respectively, using Lineweaver–Burk Plots with different concentrations of colloidal chitin (0.02–10 mg/mL), which is lower than the *K_m_* of many other chitinases [38,41]. The low *K_m_* values suggested the high affinity of MxChi to colloidal chitin.

### 3.4. Mutational Analysis

**Figure 4:**
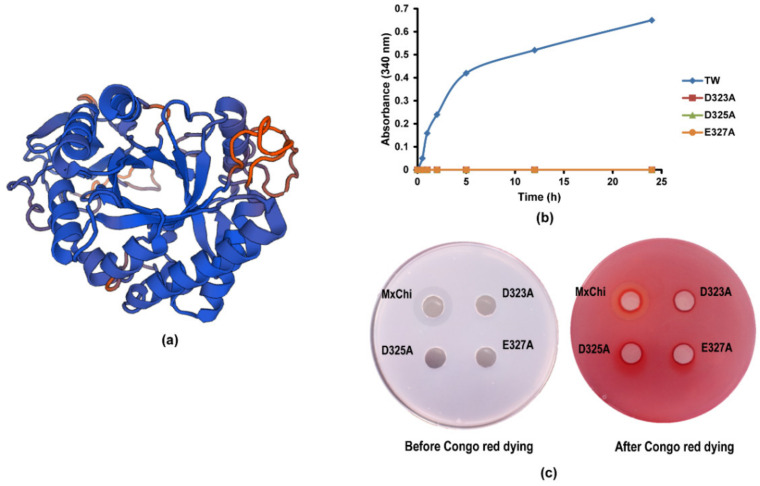
Three-dimensional homology model of MxChi and the activity screening of its mutants. (**a**) The monomer structure of MxChi obtained by homology model derived from *B. thuringiensis* (PDB ID: 6BT9). (**b**) Comparison of the activity of wild-type MxChi and its variants by microplate-reader. (**c**) Comparison of the activity of wild-type MxChi and its variants by Congo red dying. Enzyme solution was incubated in the punched hole of the media containing chitin.

The amino acid sequence alignments exhibited that the MxChi catalytic domain shared the highest sequence identity (41.9%) with chitinase from *B. thuringiensis* (PDB ID: 6BT9) (Figure S4). Therefore, 6BT9 was selected as a suitable template for MxChi three-dimensional structure building (Figure 4a). The model structure of MxChi shows that eight strands of parallel *β*-barrels are surrounded by eight *α*-helices in the core domain (named as (*β*/*α*)_8_ TIM barrel) (Figure S4), which are the typical characteristics in all GH18 chitinases with a DXDXE motif [36,50], indicating that three potential catalytic amino acids of MxChi are D323, D325, and E327. In order to evaluate the effect of these amino acid residues on the activity of MxChi, they were targeted by site-directed mutagenesis generating the single amino acid mutants D323A, D325A, and E327A. Three mutants completely lost all catalytic activity confirmed by both microplate-reader and Congo red dying methods (Figure 4b,c). Congo red-chitin complex is red color as Congo red and chitin combine [51], while the red color disappeared if the chitin in the complex was degraded. It was transparent around the wild-type MxChi treated media (Figure 4c), while the media treated by mutants of MxChi had no change in red color, implying that the mutants were inactive, which is consistent with that of the microplate-reader testing, suggesting these three amino acids are parts of the active sites. Vaaje-Kolstad et al. confirmed the D142 of ChiB in the DXDXE catalytic motif, a family 18 chitinase from *S. marcescens*, was one of the catalytic site by site-directed mutagenesis and X-ray [52]. These further verified that MxChi is a family 18 chitinase with the conserved DXDXE motif.

### 3.5. Biosynthesis of GlcN from Colloidal Chitin and Shrimp Shell

**Figure 5:**
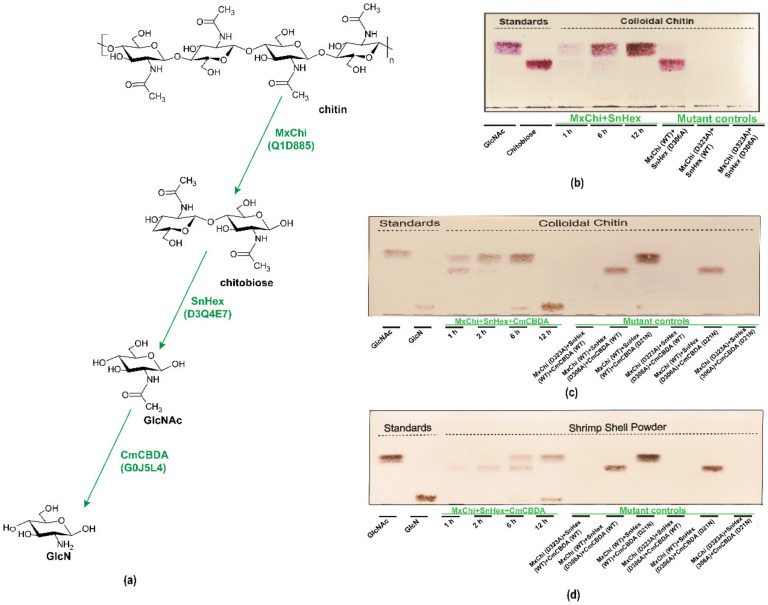
Enzymatic degradation of colloidal chitin and shrimp shell. (**a**) Schematic overview of the action of MxChi, SnHex, and CmCBDA on colloidal chitin, chitobiose and GlcNAc substrates. (**b**) TLC analysis using colloidal chitin as a substrate for the enzymatic hydrolysis to GlcNAc by MxChi and SnHex. TLC analysis using colloidal chitin (**c**) and shrimp shell (**d**) as the substrate for GlcN production by enzymatic cascades including MxChi, SnHex, and CmCBDA.

In this report, we wanted to confirm that MxChi can be used in an enzymatic cascade, in which GlcN can be produced enzymatically from chitin. Firstly, GlcNAc was generated by adding MxChi and SnHex [32] to the same reaction mixture containing colloidal chitin (Figure 5a), and the efficiency of MxChi and SnHex were successfully evaluated in a time-course reaction (Figure 5b). Only GlcNAc was detected after 12 h compared with the standard GlcNAc on TLC. Furthermore, the transparency of the reaction system was significantly increased, which means both enzymes are effective (data not shown).

Then, CmCBDA was used in the cascades for the production of GlcN from colloidal chitin and shrimp shell (Figure 5c,d) [29]. The GlcN production increased continuously over time. After a 12 h reaction time, only GlcN was detected as the colloidal chitin was the initial substrate, while some GlcNAc was still left if the initial substrate was shrimp shell, indicating that the advanced structure of destructed and homogenized colloidal chitin is easier to be approached by chitinase to elicit the further hydrolysis [53]. In the meantime, this data demonstrates that CmCBDA is not very efficient because of heat-sensitivity. 

The role of each enzyme in the hydrolysis system was further investigated by using combinations of wild-type and inactivated mutant enzymes of MxChi, SnHex, and CmCBDA (Figure 5c,d). None of the products were detected when mutant MxChi, wild-type SnHex and CmCBDA were added, which means no hydrolyzing process happened in the reaction. Chitobiose was traced when wild-type MxChi, mutant SnHex, and mutant or wild-type CmCBDA were added. GlcNAc could be detected when MxChi and SnHex were wild type in the biosynthesis system, mutant CmCBDA lost the deacetylating activity, resulting in no GlcN spot showing on the TLC. The yield of GlcN was 2.19 ± 0.34 mg from 40 mg of shrimp shell. The specificity of these enzymes made them potential candidates for pure functional saccharide production. Synergistic action of chitinase and chitosanase during fermentation for GlcN production from colloidal chitin was explored [54], while the multiple side-products and tedious purification work made it a disadvantage. Several chitinolytic enzymes were explored for the enzymatic degradation of chitin [42,45]. However, most of research focused on GlcNAc or chitooligomers production [37,38]. Few studies about the biocatalytic production of GlcN from chitin were reported [55].

### 3.6. Single Factor Test Results

**Figure 6:**
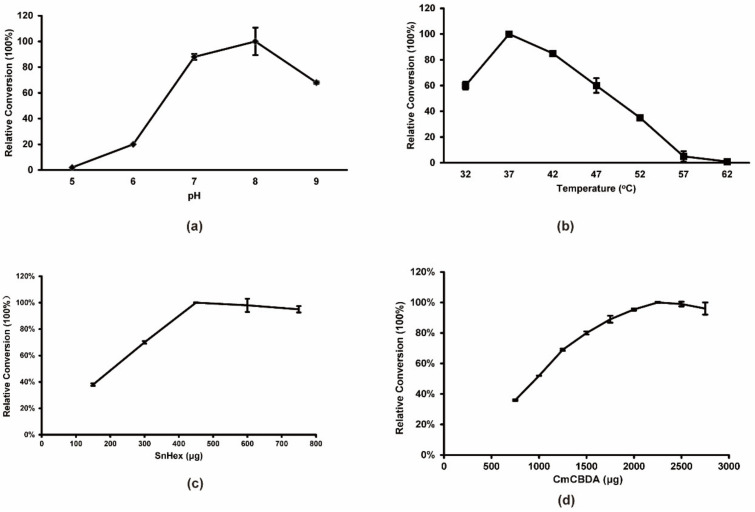
Single factor test result for the production of GlcN from shrimp shell. (**a**) Effect of pH on enzymatic cascades for the production of GlcN. (**b**) Temperature influence on the enzymatic cascades for the production of GlcN. (**c**) The optimum enzyme amount ratio of MxChi to SnHex. (**d**) The optimum enzyme amount ratio of MxChi/SnHex to CmCBDA. The error bars represent the standard deviations calculated from three independent experiments.

Orthogonal design is an important and efficient statistical method, which makes the effects of several factors with two or more levels on a response, to be conducted in a relatively small number of runs [56,57,58]. In the present work, the effect of different pH on the production of GlcN from shrimp shell is shown in Figure 6a. Though the optimal pH of MxChi was 7.0, the highest conversion appeared when the pH was 8.0 for enzymatic cascades. The temperature was a significant factor for the biosynthesis of GlcN. The production of GlcN increased as the temperature enhanced from 32 °C to 37 °C, while the conversion decreased over 37 °C (Figure 6b). 37 °C was the optimum temperature in the single factor test. The initial ratio of MxChi/SnHex/CmCBDA was 1/1/5 (*w*/*w*/*w*), and the amount of MxChi was 100 μg. Figure 6c showed that the production increased as the amount of SnHex increased from 150 to 450 μg, and slightly decreased with a higher amount of SnHex. Considering the applicability, 450 μg of SnHex was chosen as the optimal amount. Based on the best ratio of MxChi to SnHex (1:3), a different amount of CmCBDA was tested. CmCBDA was the third enzyme in the enzymatic trace. More quantity of CmCBDA had to be used to acquire more production of GlcN. Poor thermostability of CmCBDA should also be taken into account. The optimum amount of CmCBDA was 2250 μg showing in Figure 6d. The best ratio was 1/3/15 of MxChi/SnHex/CmCBDA. Therefore, the factor levers for enzyme amount were shown in Table 1.

### 3.7. Optimization of GlcN Biosynthesis by Orthogonal Design

In this study, orthogonal array L9 (3^3^) containing three factors and three different levels for each factor was performed to improve the production of GlcN, shown in Table 1. Both the main effect analysis and ANOVA methods were utilized to compare the effect of different factors of enzymatic cascades on the production of GlcN from chitin, and the experimental results of the orthogonal design are shown in Table 2 and Table 3. The processing factors resulting in GlcN production from 40 mg shrimp shell were 0.45–2.48 mg. In spite of the significant contribution of these factors, the reaction temperature (**B**) showed the greatest influence on the production of GlcN, followed by reaction of pH (**A**) and enzyme amount ratio (**C**). The optimal combination was **A_2_B_2_C_3_**, the optimum conditions for the biosynthesis of GlcN from the shrimp shell were the reaction at pH 8.0, temperature at 37 °C, and the enzyme amount ratio of MxChi/SnHex/CmCBDA at 1/3/16.5. 

According to this conclusion, the optimal condition was not shown in the nine orthogonal experiments. Furthermore, orthogonal design has yet to be applied to the optimization of GlcN biocatalytic production from chitin. A verification test of the orthogonal experiment in this report was carried out by three parallel enlarged enzymatic cascade reaction systems (400 mg shrimp shell) under optimum condition, and 26.33 ± 1.24 mg of GlcN was quantified by microplate-reader. The GlcN yield was a little higher than the highest value in the orthogonal test. A combination with the application of orthogonal design on the biosynthesis of other compounds [59] indicated that the orthogonal array for the optimization of GlcN biotransformation was available. The yield of GlcN hydrolyzed from shrimp shell increased 20.09% with the optimized condition. Immobilized chitinolytic enzymes for optimizing the biocatalytic production of GlcN from colloidal chitin were explored by Bao et al. [55]. Concentrated hydrochloric acid is usually employed to crustacean shell and the chitin source for industrial preparation of GlcN [13,60]. However, this method has the disadvantages of low yield [61] environmental pollution, and a high content of sodium chloride in the final product, which may increase the cost and risk of some diseases, such as heart failure, osteoporosis, and kidney disease [62]. Alternatively, our report may also provide the possibility for non-animal source consumption such as mushrooms [29], in considering the consumers with crustacean allergies and vegan diets. 

## 4. Conclusions

A novel chitinase from *M. xanthus* was discovered and cloned successfully. MxChi could be well overexpressed in soluble and biochemically characterization. It exhibited maximum activity at 55 °C, pH 7, and especially robust below 37 °C. Multiple detection technologies confirmed that this recombinant enzyme hydrolyzes colloidal chitin to chitobiose as sole product. Enzymatic cascades containing specificity enzymes of chitinase MxChi, *β*-N-acetylhexosaminidase and GlcNAc deacetylase, could efficiently convert chitin or shrimp shell from biomass into high-value dietary supplement GlcN under the optimized condition. Enzymatic cascade reaction demonstrates significant potential for large scale production of GlcN, while genetic manipulation for co-expression of three genes encoding these three enzymes and the thermostability modification of CmCBDA could be attempted to enhance the catalytic efficiency in order to augment their commercial competence. The high performance of this reported MxChi makes it powerful for the green industrial conversion of chitinous waste to a high-value product for the food industry and future research. 

## Figures and Tables

**Table 1:** Level and factor of orthogonal design.

Level	Factors		
	pH(A)	Temperature(B)	Enzyme Amount Ratio (C) (MxChi: SnHex: CmCBDA, μg)
1	7	32	150: 450: 2000
2	8	37	150: 450: 2250
3	9	42	150: 450: 2500

Symbols A, B, and C represent factors of enzymatic pH, enzymatic temperature, and enzyme amount ratio. Symbols 1, 2, and 3 represent concentration levels of each factor.

**Table 2:** A L9 (3 ^3^) orthogonal array and experimental results.

Exp. Number	Factors			Product (mg)
	pH(A)	Temperature(B) (℃)	Enzyme Amount Ratio (C) MxChi: SnHex: CmCBDA, μg)	
1	7	32	150: 450: 2500	0.96
2	7	37	150: 450: 2250	2.02
3	7	42	150: 450: 2000	1.57
4	8	32	150: 450: 2250	0.88
5	8	37	150: 450: 2000	2.48
6	8	42	150: 450: 2500	1.99
7	9	32	150: 450: 2000	0.45
8	9	37	150: 450: 2500	1.67
9	9	42	150: 450: 2250	1.21
K_1_	4.55	2.29	4.50	
K_2_	5.35	6.17	4.11	
K_3_	3.33	4.77	4.62	
$\bar{k1}$	1.51	0.76	1.50	
$\bar{k2}$	**1.78**	**2.06**	1.37	
$\bar{k3}$	1.11	1.59	**1.54**	
R	0.76	1.30	0.17	
**Best level**	**A_2_B_2_C_3_**			

The arrangements of column **A**, **B**, and **C** were decided by orthogonal design for 3 (factor) * 9 (run number); every row of run number represents one experimental replicate.

**Table 3:** Results of variance analysis.

Source of Variation	Sum of Square	Variance	Mean Square	F-Value	*p*-Value	Significant
Factor **A**	0.41	1	0.41	32.03	0.0008	**
Factor **B**	1.17	1	1.17	91.54	<0.0001	***
Factor **C**	0.18	1	0.18	14.31	0.0069	**
Error	0.038	4	9.57 × 10^−3^			
Sum	1.798	7				

**, *** and Blank represent more significant different, significant different and no significant different, respectively.

## Supplementary Materials

The following are available online at https://www.mdpi.com/article/10.3390/foods10112808/s1, Figure S1. Agarose electrophoresis of the amplified MxChi DNA fragment. Figure S2. Microscopic changes of MxChi treated colloidal chitin in different time interval. Figure S3. Schematic overview of the experimental setup for the microplate reader-based spectrophotometric detection of MxChi activity. Figure S4. Homology alignment of MxChi with chitinase homologues from Serratia marcescens (Sm), Streptomyces thermoviolaceus (St), Bacillus thuringiensis (Bt), and Aspergillus fumigatus (Af). Table S1 Primers for site-directed mutation.
