# Supplementary material for: Heterologous Expression of a Thermostable Chitinase from Myxococcus xanthus and Its Application for High Yield Production of Glucosamine from Shrimp Shell
Source: Foods. 2021 Nov 15;10(11):2808. doi: 10.3390/foods10112808 (PMC8619855; doi:10.3390/foods10112808)
Supplement: Supplementary file 1 [file foods-10-02808-s001.zip › foods-1391402-Supplementary information.pdf]

foods

MDPI

*Article*

# Heterologous Expression of a Thermostable Chitinase from *Myxococcus xanthus* and Its Application for High Yield Production of Glucosamine from Shrimp Shell

**Yongmei Lyu [1,†], Feng Zheng [2,†], Chuanxing Qiu [1], Meng Wang [2], Dujun Wang [1], Xiaoyang Zhang [1], Josef Voglmeir [2], Li Liu [2,*] and Xiaohong Yu [1,*]**

[1] School of Marine and Bioengineering, Yancheng Institute of Technology, Yancheng 224051, China; lyu.yongmei@ycit.edu.cn (Y.L.); xq19516560569@163.com (C.Q.); wangdj@ycit.edu.cn (D.W.); zhang.xiaoyang@ycit.edu.cn (X.Z.)

[2] Glycomics and Glycan Bioengineering Research Center (GGBRC), College of Food Science and Technology, Nan-jing Agricultural University, Nanjing 210095, China; 2016108004@njau.edu.cn (F.Z.); 2016208028@njau.edu.cn (M.W.); josef.voglmeir@njau.edu.cn (J.V.)

* Correspondence: lichen.liu@njau.edu.cn (L.L.); yxh1127@ycit.edu.cn (X.Y.); Tel.: +86-25-8439-9553 (L.L.); +86-515-8829-8960 (X.Y.)

† All authors contributed equally.

**Citation:** Lyu, Y.; Zheng, F.; Qiu, C.; Wang, M.; Wang, D.; Zhang, X.; Voglmeir, J.; Liu, L.; Yu, X. Heterologous Expression of a Thermostable Chitinase from *Myxococcus xanthus* and Its Application for High Yield Production of Glucosamine from Shrimp Shell. *Foods* **2021**, *10*, 2808. https://doi.org/10.3390/foods10112808

Academic Editors: Nabil Grimi and Gianpiero Pataro

Received: 7 September 2021
Accepted: 10 November 2021
Published: 15 November 2021

**Publisher's Note:** MDPI stays neutral with regard to jurisdictional claims in published maps and institutional affiliations.

**Copyright:** © 2021 by the authors. Submitted for possible open access publication under the terms and conditions of the Creative Commons Attribution (CC BY) license (https://creativecommons.org/licenses/by/4.0/).

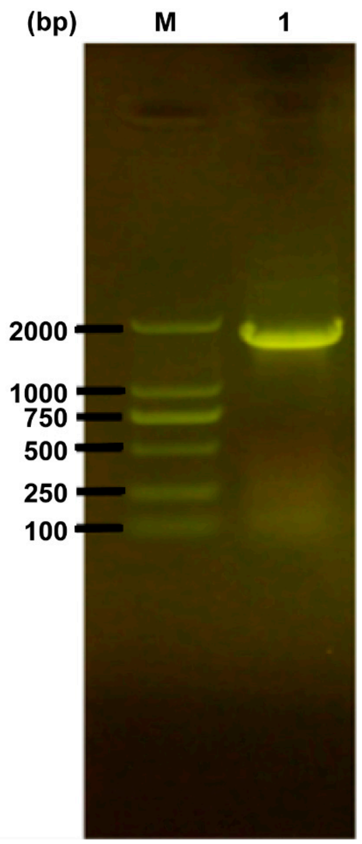


**Figure S1.** Agarose electrophoresis of the amplified MxChi DNA fragment. M: DNA ladder, 1: PCR product of MxChi (theoretical length is 1731 bp).

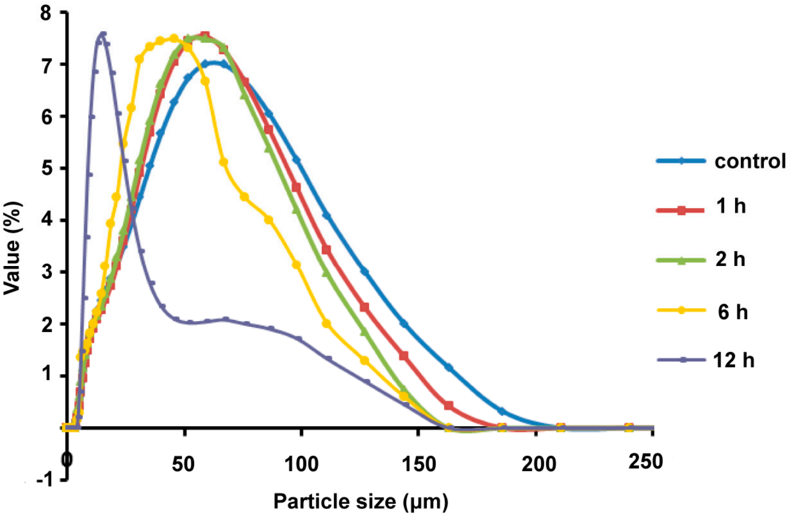


**Figure S2.** Microscopic changes of MxChi treated colloidal chitin in different time interval.

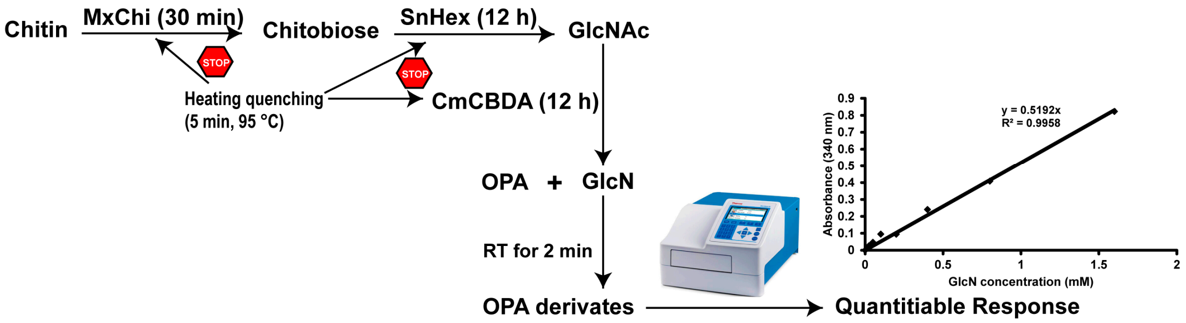


**Figure S3.** Schematic overview of the experimental setup for the microplate reader-based spectrophotometric detection of MxChi activity.

```
Q1D885 Mx : -----------------------------------------------------------------VPSSHVRDMTAFQDA :  15
P11797 Sm : MSTRKAVIGYYFIPTNQINNYTETDTSVVPFPVSNITPAKAKQLTHINFSFLDINSNLECAWDPATNDAKARDVV----- :  75
D6K143 St : -------------------------------------------------------------------------------- :   -
Q81IF9 Bt : -------------------------------------------------------------------------------- :   -
Q873X9 Af : -------------------------------------------------------------------------------- :   -

Q1D885 Mx : ALAAEWAAGVAYSVGTRVTYQGRLYECRQPHTSQADWTPVAVASLWLDLGPAGGGEPDAGSGGTDAGTGGDVTAPTVGLS :  95
P11797 Sm : -------------------------------------------------------------------------------- :   -
D6K143 St : -------------------------------------------------------------------------------- :   -
Q81IF9 Bt : -------------------------------------------------------------------------------- :   -
Q873X9 Af : -------------------------------------------------------------------------------- :   -

Q1D885 Mx : ASASRIIAVGPLSLTATATDDVGVTRVEILENGAVVATGSQFSRAFSGWEQNG---TYVYAVRAYDAAGNVGTTTLTVVV : 172
P11797 Sm : -------------------------------------------------------------------------------- :   -
D6K143 St : --------------------------------------------------MKYLLPTAAAGLLLLAAQPAMAMATDHSPT :  30
Q81IF9 Bt : --------------------------------------------------MNH---K-VHHHHHHIEGRHMELGTLEGSE :  26
Q873X9 Af : -------------------------------------------------------------------------------- :   -

Q1D885 Mx : EIPGGPPPGGKRIVGYFTAWGIYARNYHVSNVQPS----KLTHINYAFSNISGD-GR----------------------- : 224
P11797 Sm : -------------------------------------------------------------------------------- :   -
D6K143 St : VETRAAADNGTVKLGYFTEWGTYDRNFNVKNLDTSGTAAKITHINYAFGNVTG--GK----------------------- :  85
Q81IF9 Bt : FKLMDSPKQSQKIVGYFPSWGVYGRNYQVADIDAS----KLTHLNYAFADICWN-GKHGNPSTHPDNPNKQTWNCKESGV : 101
Q873X9 Af : ------ASSGYRSVVYFVNWAIYGRNHNPQDLPVE----RLTHVLYAFANVRPETGE----------------------- :  47

Q1D885 Mx : -----------CILGDPFADIDKSG----GWQGE---W-DPGQL-R-GNFRAFKEMKRQNPHLKLLISVGGWSWST---- : 279
P11797 Sm : --------------------------------------------------NRLTALKAHNPSLRIMFSIGGWYYSNDLGV : 105
D6K143 St : -----------CAIGDSYADYDKAFTADQSVSGQADTW---DQPLR-GNFNQLRQLKAKYPHIKVLWSFGGWTWSG---- : 146
Q81IF9 Bt : PLQNKEVPNGTLVLGEPWADVTKSY----PVSGT--TWEDCDKYARCGNFGELKRLKAKYPHLKTIISVGGWTWSN---- : 171
Q873X9 Af : -----------VYMTDSWADIEKHY------PGD--SWSDTGNNVY-GCIKQLYLLKKQNRNLKVLLSIGGWTYSP---- : 103

Q1D885 Mx : ---HFSTVASSPASRAAFVKSCVDLYIRGQYPGVDPVNGEGVFDGIDIDWEYPVGGGLPGNSNSPADKQNYTLLMQEFRS : 356
P11797 Sm : SHANYVNAVKTPASRAKFAQSCVRIMKDYG------------FDGVDIDWEYP----------QAAEVDGFIAALQEIRT : 163
D6K143 St : ---GFADAAKDPQG---FAQSCYNLVHDPRW--------DGVFDGIDIDWEYPNACGLTCDSSGPDAFRN---LMAALRS : 209
Q81IF9 Bt : ---RFSDMAADEKTRKVFAESTV-AFLRA-Y-------G---FDGVDLDWEYPGVETIPGGSYRPEDKQNFTLLLQDVRN : 236
Q873X9 Af : ---NFAPAASTDAGRKNFAKTAVKLLQDLG------------FDGLDIDWEYP---------ENDQQANDFVLLLREVRT : 159

Q1D885 Mx : QLNAVTTQT---GKPYLLTIATGASPDLLENK-QETKKLSDVLDWINVMSYDYHGAFEST--VNFHSALHRVTGDP---- : 426
P11797 Sm : LLNQQTITDGRQALPYQLTIAGAGGAFFLSRYYSKLAQIVAPLDYINLMTYDLAGPWEKV--TNHQAALFGDAAGPTFYN : 241
D6K143 St : TFGDELVTA---A----VT-ADGTPGGKIE-A-TDYAGAAQYVDWYNVMTYDFFGAWDAQGPTAPHSPLTSYDGIP---- : 275
Q81IF9 Bt : ALNKAGAED---GKQYLLTIASGAS-QRYADH-TELKKISQILDWINIMTYDFHGGWEAT--SNHNAALYKDPNDP---- : 305
Q873X9 Af : ALDSYSAANAG-GQHFLLTVASPAGPDKIK-V-LHLKDMDQQLDFWNLMAYDYAGSFSSL--SGHQANVYNDTSNP---- : 230

Q1D885 Mx : ----------------GAATGF--YTDGSVSKMLA-LGVPPAKIVVGVPFYGRGWGSVPNVNNGLFQS-GVP-------T : 479
P11797 Sm : ALREANLGWSWEELTRAFPSPFSLTVDAAVQQHLMMEGVPSAKIVMGVPFYGRAFKGVSGGNGGQYSSHSTPGEDPYPST : 321
D6K143 St : ------------------KQGF--TSADAIAAFKA-QGVPADKLLLGIGFYGRGW-------TGVTQD-A-PGGTATGPA : 325
Q81IF9 Bt : ----------------AANTNF--YVDGAINVYTN-EGVPVDKLVLGVPFYGRGWKSCGKENNGQYQP-CKPGSDGKLAS : 365
Q873X9 Af : -----------------LSTPF--NTQTALDLYRA-GGVPANKIVLGMPLYGRSFANT----DG-------PGKPYNGVG : 279

Q1D885 Mx : R----GTWDDGSSGLTGVFDFKDIKANYERPGSGYTKFFHPEAKEAYVYNPATGIWIGYDDVQSINAKADYILNKNLGGA : 555
P11797 Sm : DYWLVGCEECVRDKDPRIASYRQLE-QMLQGNYGYQRLWNDKTKTPYLYHAQNGLFVTYDDAESFKYKAKYIKQQQLGGV : 400
D6K143 St : A----GTWEQ------GIEDYKVLK-N-TCPVTGTV------AGTAYAH-CGSNLW-SYDTPDTIASKMAWANDQGLRGA : 385
Q81IF9 Bt : K----GTWDDYSTGDTGVYDYGDLAANYVNK-NGFVRYWNDTAKVPYLYNATTGTFISYDDNESMKYKTDYIKTKGLSGA : 440
Q873X9 Af : Q----GSWEN------GVWDYKAL------PQAGATEHVLPDIMASYSYDATNKFLISYDNPQVANLKSGYIKSLGLGGA : 343

Q1D885 Mx : MFWELSGD------------------------------------------------------------------------ : 563
P11797 Sm : MFWHLGQD------------------------------------------------------------------------ : 408
D6K143 St : FAWDFSGD------------------------------------------------------------------------ : 393
Q81IF9 Bt : MFWELSGDCRTSPKYSCSGPKLLDTLVKELLGGPINQKDTEPPTNVKNIVVTNKNSNSVQLNWTASTDNVGVTEYEITAG : 520
Q873X9 Af : MWWDSSSD------------------------------------------------------------------------ : 351
```

**Figure S4.** Homology alignment of MxChi with chitinase homologues from Serratia marcescens (Sm), Streptomyces thermoviolaceus (St), Bacillus thuringiensis (Bt), and Aspergillus fumigatus (Af). The aspartic acid and glutamic acid residues highlighted in red are the proposed catalytic amino acids. Uniprot identifiers are shown with the abbreviation of species.

**Table S1.** Primers for site-directed mutation.

| Gene or Mutation Site | Primer | Sequence |
|---|---|---|
| D323A | F3 | 5'-GTGTTCGACGGCATCGCCATCGACTGGGAGTACCCGGTCGGCGG-3' |
| | F4 | 5'- CCGCCGACCGGGTACTCCCAGTCGATGGCGATGCCGTCGAACA-3' |
| D325A | F5 | 5'-TTCGACGGCATCGACATCGCCTGGGAGTACCCGGTCGGCG -3' |
| | F6 | 5'-CGCCGACCGGGTACTCCCAGGCGATGTCGATGCCGTCGAA -3' |
| E327A | F7 | 5'-GGCATCGACATCGACTGGGCCTACCCGGTCGGCGGCGGCC -3' |
| | F8 | 5'-GGCCGCCGCCGACCGGGTAGGCCCAGTCGATGTCGATGCC -3' |

Note: Mutant sites are underlined with solid line.
